# Large-Sized Bilateral Atherosclerotic Axillary Artery Aneurysms: A Case Report

**DOI:** 10.5334/jbr-btr.968

**Published:** 2016-01-28

**Authors:** Aysegul Idil Soylu, Ahmet Veysel Polat

**Affiliations:** 1 Ondokuz Mayis University Medical Faculty, TR

**Keywords:** giant axillary artery aneurysm, atherosclerosis

## Abstract

Axillary artery aneurysms are most commonly caused by trauma, while atraumatic cases are very rare. Atherosclerosis, collagen tissue diseases, and mycotic infections are additional etiological factors for this type of aneurysms. Once developed, an aneurysm poses risk for hand ischemia or neurologic complications due to embolic events and neural compression. Due to this, diagnosis and treatment of an aneurysm are crucial. We report a case of giant bilateral axillary artery aneurysms developed as a consequence of atherosclerosis.

## Introduction

Axillary artery aneurysms are the peripheral artery aneurysms that are seen quite rarely [1,2]. They are usually reported to develop secondary to upper extremity trauma. Collagen tissue diseases, atherosclerosis, and thoracic outlet syndrome are the other etiologies of axillary artery aneurysms. The condition is usually asymptomatic; however, some patients may have symptoms such as extremity pain, numbness, and hand ischemia. In this report, we present a case of a patient who had complaint of masses in the axillae that were due to giant atraumatic bilateral axillary artery aneurysms.

## Case Report

**Figure 1:**
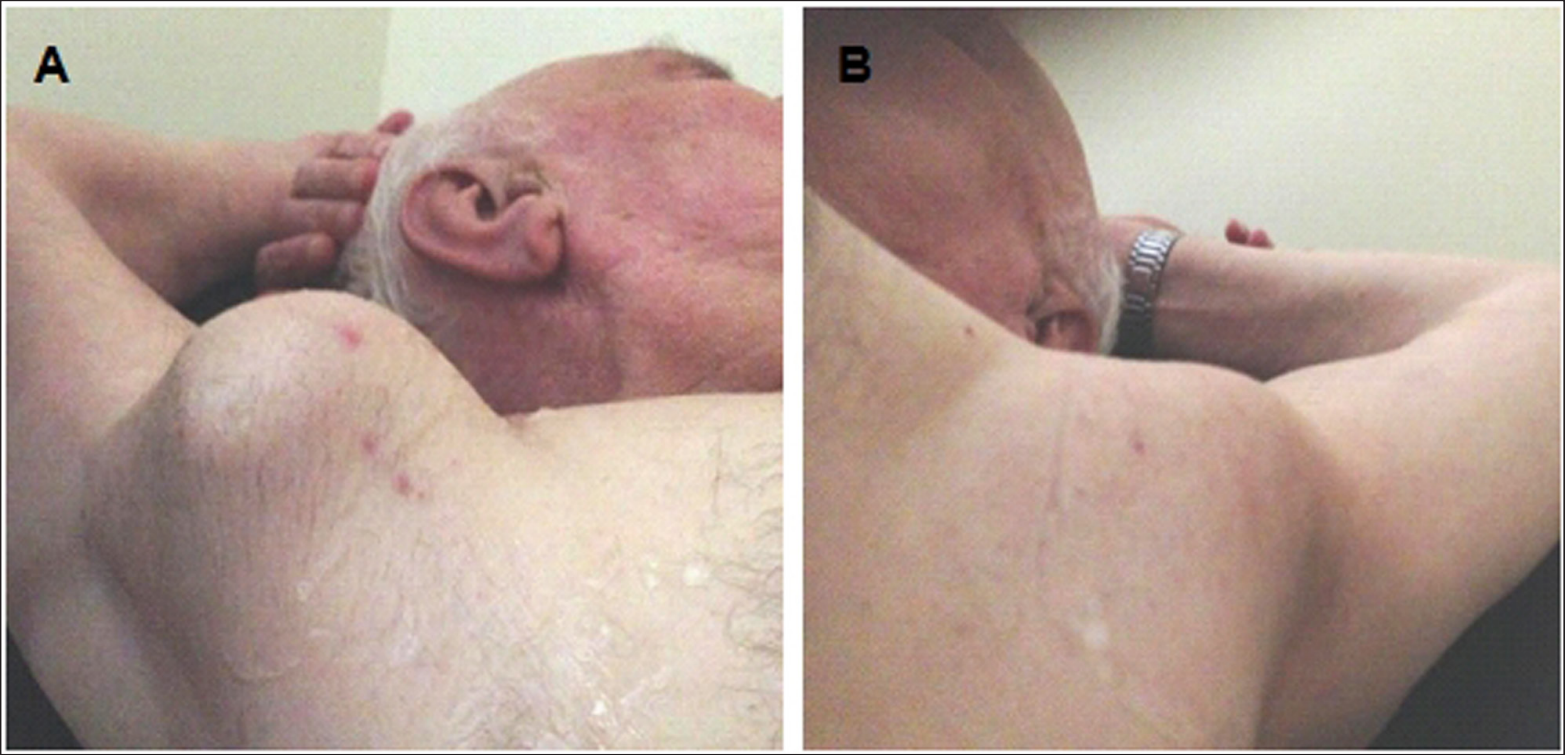
External appearance of the bilateral axillary pulsatile masses. (A) right, (B) Left.

**Figure 2:**
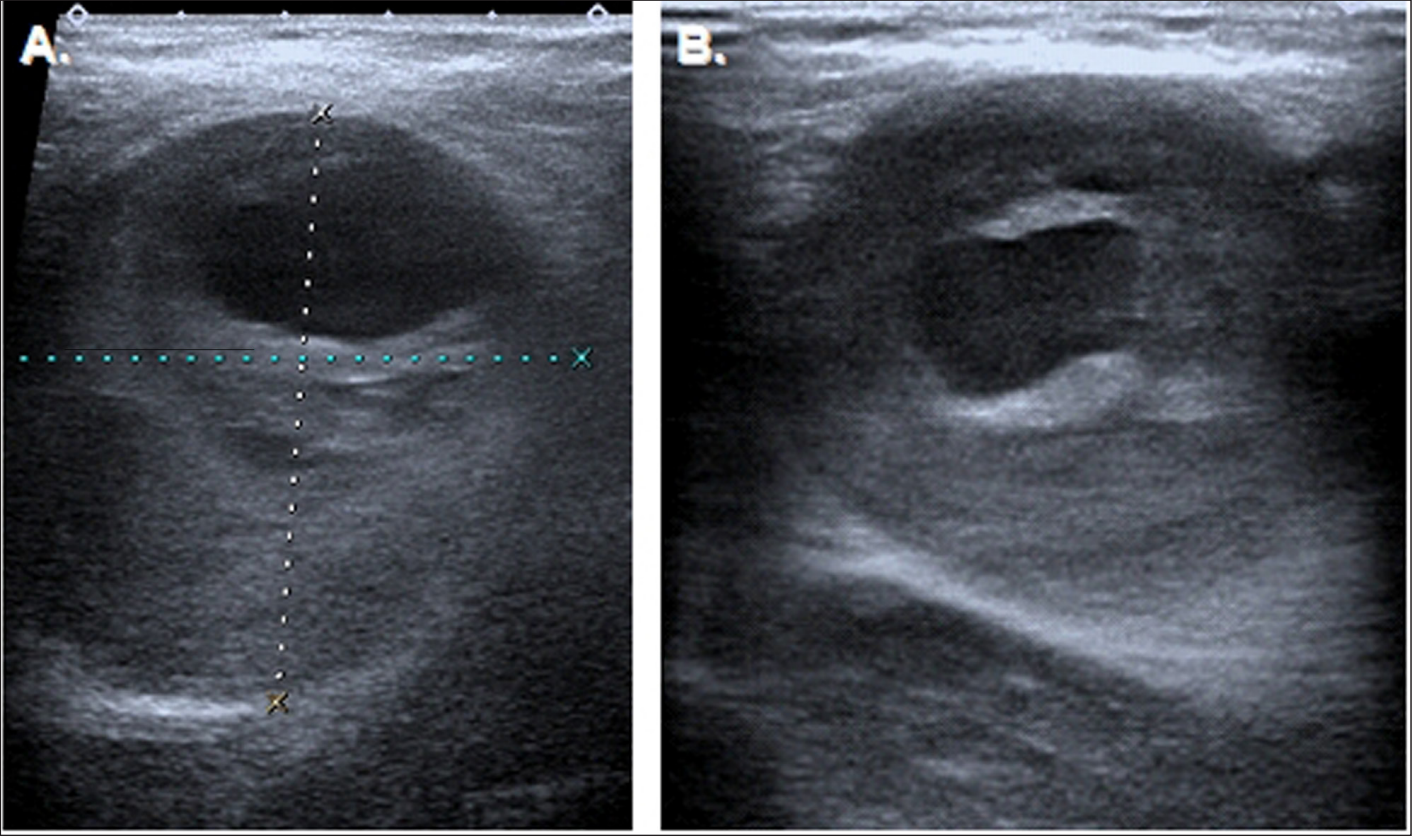
Ultrasonography of the axillary masses. (A) Transverse section show 67 × 45 mm partially thrombosed aneurysm on the right, (B) 40 × 45 mm partially thrombosed aneurysm on the left.

**Figure 3:**
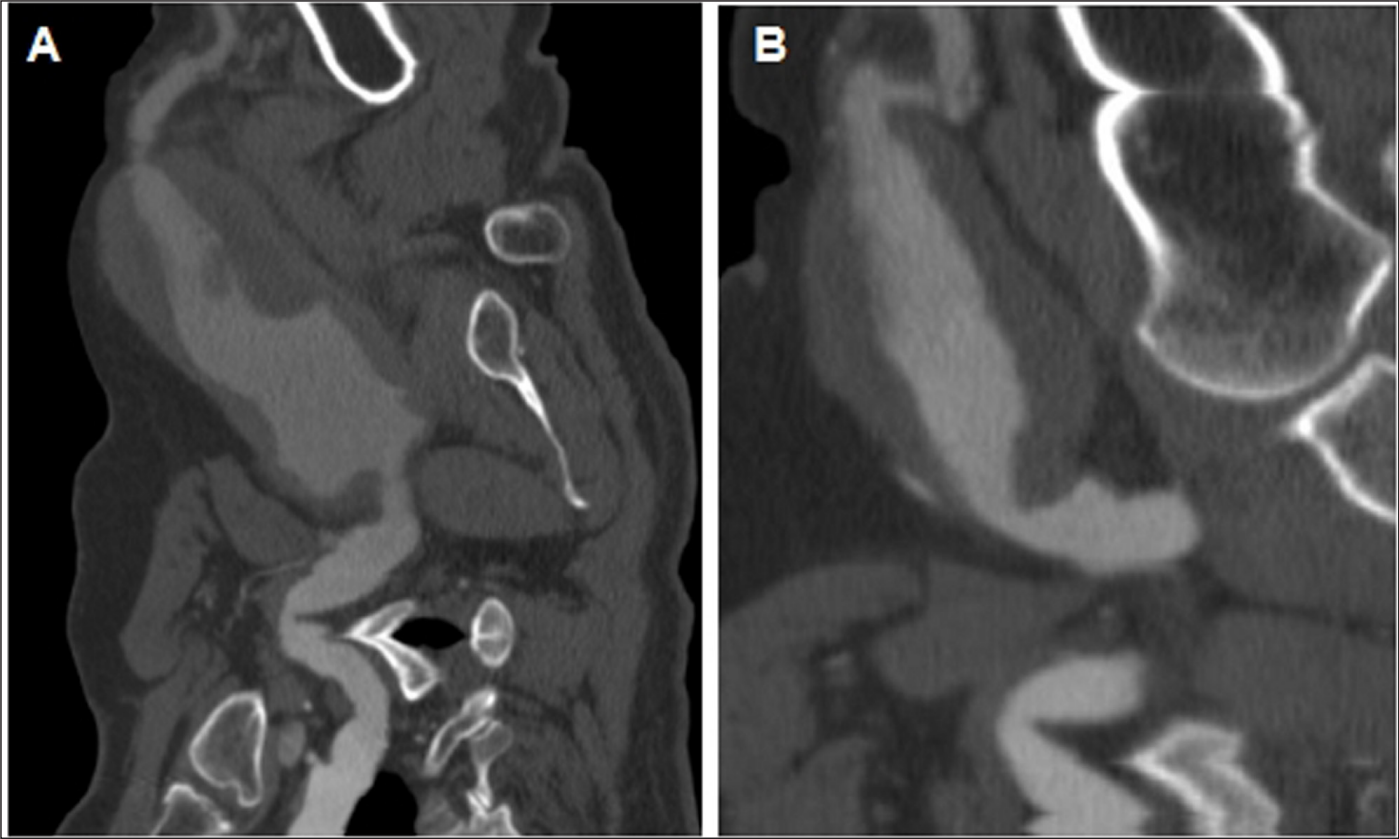
On a CT angiogram, bilateral axillary artery aneurysms were detected on curvilinear reformatted sections. (A) right, (B) Left.

The 72 year old male patient was referred to our clinic with the complaint of bilateral axillary masses and numbness in the right hand. In his medical history, he had hypertension for 4 years but no history of trauma. A physical examination showed the presence of palpable pulsatile masses in his both axillae (12 cm in size in the right axilla and 8 cm in size in the left axilla) (Fig. 1). His neurological and systemic examinations were within normal limits. An upper-extremity ultrasound examination revealed an aneurysm in a 12-cm segment of the right axillary artery, reaching 67 × 45 mm in dimension. There was a 27-mm thick thrombus at the edge of the aneurysm lumen. Adjacent to the aneurysm, there was a 48 × 40 mm hyperechogenic mass not related to the aneurysm. There was also an aneurysm reaching 40 × 43 mm in dimension in a 9-cm segment of the left axillary artery, with a 17-mm thick thrombus. A Doppler ultrasonic examination revealed a thrombus surrounding the lumen and a turbulent, pulsatile flow in the center. The aneurysmal segment was continuous with the brachial artery. There was no blood flow in the region adjacent to the right axillary artery (Figs. 2 and 3). On CT angiography, giant fusiform aneurysms were detected, measuring 140 × 77 mm in the right axillary artery and 93 × 45 mm in the left axillary artery. There were thrombi in both aneurysms, allowing the flow in the lumen. There was also an extension to the brachial artery and contour irregularities at the distal end of the right-side aneurysm. These latter findings were proposed to be due to a spontaneously healed rupture. A physical examination of the eyes, bones, skin and joints revealed no signs of collagen tissue disease, nor was it detected through laboratory examinations. Because the patient was symptomatic and had a high risk of aneurysm rupture, endovascular closure was initially planned. However, appropriate size stents could not be found for aneurysms so large and tortuous. Besides, the long-term success of the stenting procedure could not be guaranteed due to the large sizes and high mobility of the aneurysms, so the patient was referred for surgery.

## Discussion

An axillary artery aneurysm is a very rare clinical condition, comprising 0.5% to 1% of all peripheral arterial aneurysms [1]. It usually develops secondary to blunt or penetrating trauma [1]. Other etiologies are collagen tissue disease, atherosclerosis, mycotic infections, and thoracic outlet syndrome [3]. The majority of the axillary artery aneurysms develop secondary to the use of crutches or to sportive activities in which the arms are actively used and subjected to repetitive blunt microtraumas. Repetitive traumas may lead to endothelial dysfunction which plays a role in the pathogenesis of axillary artery aneurysms [4,5,6]. Atraumatic axillary artery aneurysms secondary to atherosclerosis are rarely seen, and the patients are usually asymptomatic. Our patient did not have any history of trauma or surgery of the axillary region, nor did he have thoracic outlet syndrome or a mycotic infection. His physical and laboratory examinations did not reveal collagen tissue disease either. For these reasons, the aneurysms of our patient were considered to be a consequence of atherosclerosis.

In the literature, axillary artery aneurysms are mostly unilateral. Bilateral axillary artery aneurysms were only reported in two Marfan syndrome patients [7,8]. However, thrombus in the aneurysmal lumen, which are used for differential diagnosis of atherosclerotic aneurysms, were not reported in either of the Marfan syndrome patients [3]. Therefore, thrombus in the aneurysm suggests atherosclerotic etiology in our patient. Our patient is the first patient reported to have bilateral axillary artery aneurysms secondary to atherosclerosis. In addition, the largest axillary artery aneurysm previously reported in the literature was 103 × 45 mm, so our case represents the largest axillary artery aneurysm ever reported.

Surgery is the standard treatment for axillary artery aneurysms; however, endovascular methods have become more commonly used in recent years due to emerging techniques and equipment [2,10,11]. Graft stents have become the procedure of choice for the endovascular treatment of peripheral aneurysms [9]. However, stent placement near a mobile joint carries the risk of stent deformation or breaking. The largest aneurysm treated by an endovascular method was 3 × 5 cm in size. Surgery is preferred for larger and tortuous aneurysms [7]. We also opted for surgery in this case because the aneurysms were very large and tortuous.

## Conclusion

Bilateral axillary artery aneurysms are a very rare clinical condition detected as masses in the axillary region. Detailed examination of the nature and size of the aneurysm with imaging techniques is crucial in treatment planning.

## Competing Interests

The authors declare that they have no competing interests.
